# Supplementary material for: Texture is encoded in precise temporal spiking patterns in primate somatosensory cortex
Source: Nat Commun. 2022 Mar 14;13:1311. doi: 10.1038/s41467-022-28873-w (PMC8921276; doi:10.1038/s41467-022-28873-w)
Supplement: Supplementary file 1 — Supplementary Information [file 41467_2022_28873_MOESM1_ESM.pdf]

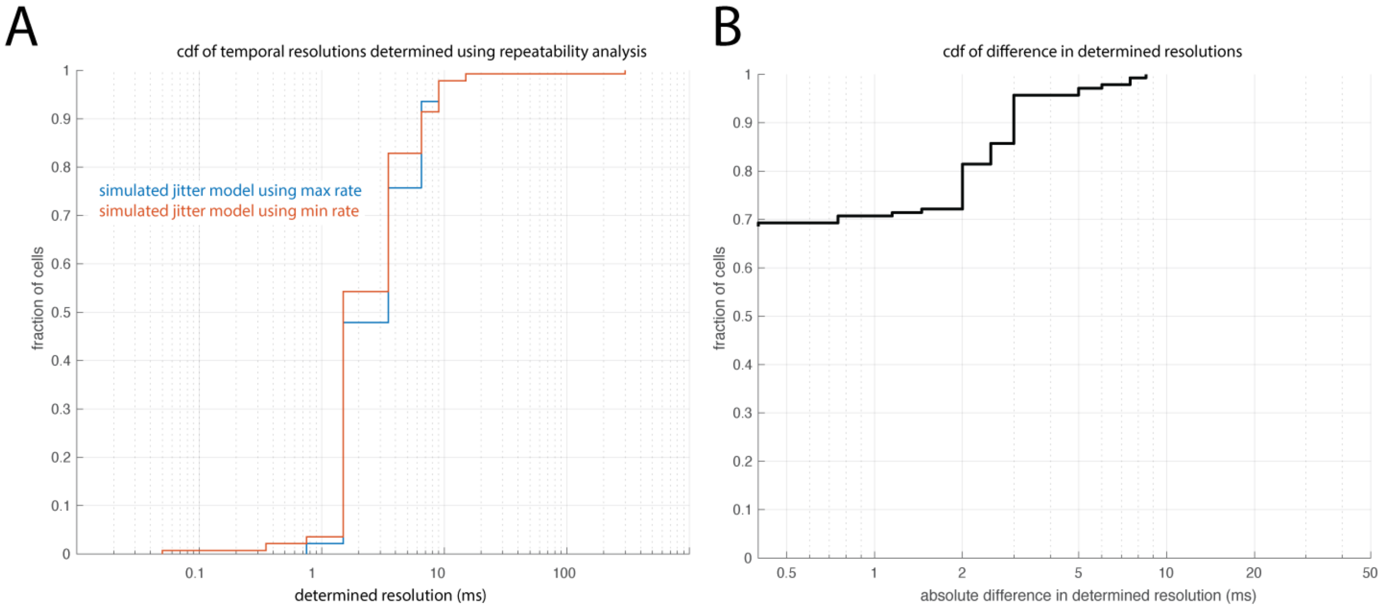

Supplemental Figure 1: Different temporal resolutions determined using each jitter model are the same. A| We generated our simulated jitter models for each cell and texture using the trial with the largest number of spikes (max rate, blue) or the smallest (min rate, red). We then found the determined resolution for each cell (n = 141) using these two jitter models. The determined resolutions were indistinguishable using each these two models (max and min rate, compared using two-sided, two-sample Kolmogorov-Smirnov test; D = .06, p = .93). Here, each line represents the cumulative distribution function of temporal resolutions of all cortical cells using each method (max rate in blue, min rate in red). B| Cumulative distributions of the absolute difference in temporal resolution determined using each model. More than 90% of cells have resolutions that differ by 3 ms or less.

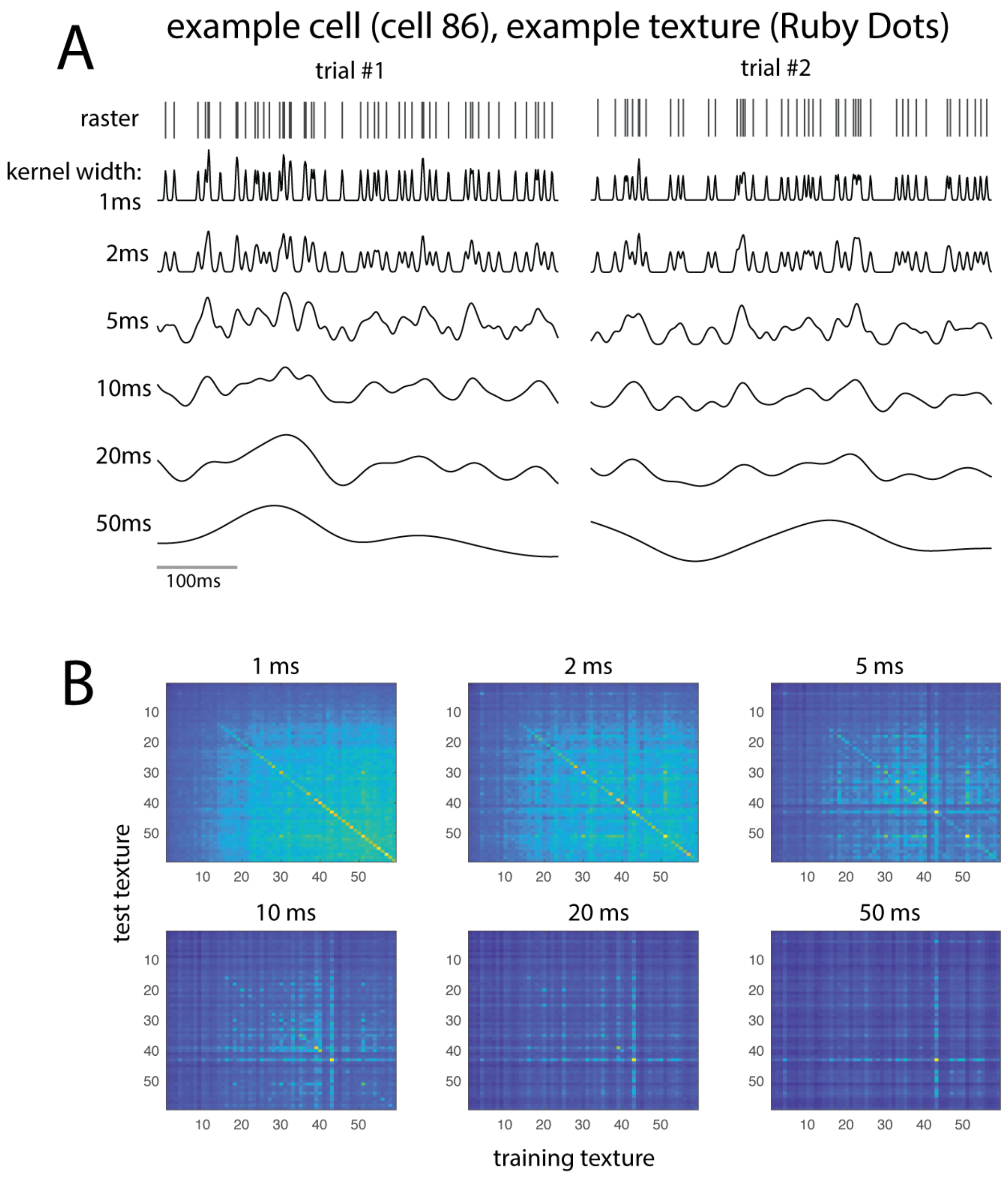

Supplemental Figure 2: Timing-based classification method. A| Spike times for two trials of a given example cell and example texture, and the same trials' PSTHs generated using smoothing kernels of varying widths (1 ms to 50 ms). B| Heat maps showing the max cross-correlation values for every pair of textures. Textures are sorted by mean firing rate for the given example cell. Heat maps are shown using PSTHs at each temporal resolution depicted in A (1ms-50ms, blue - yellow).

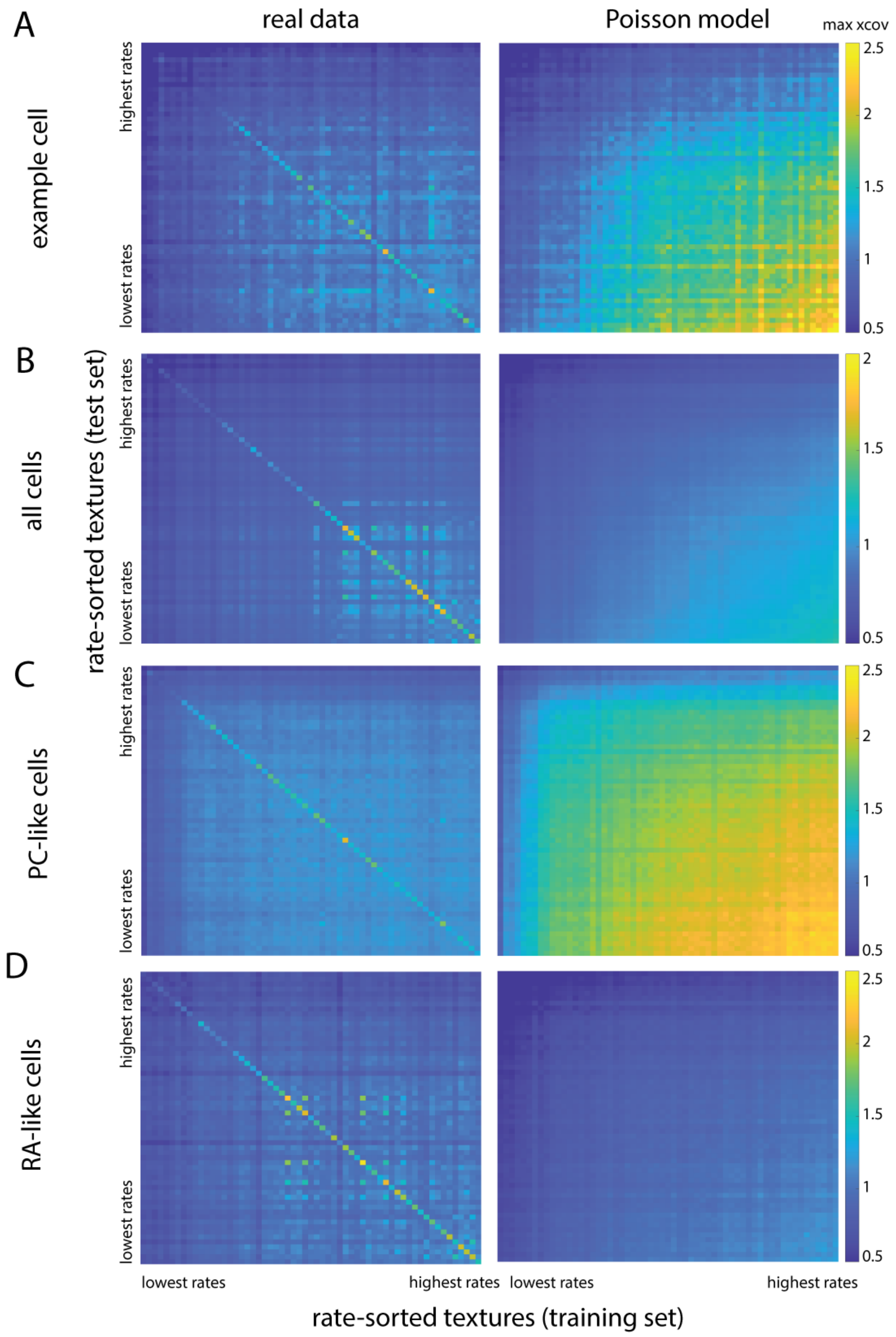

Supplemental Figure 3: Confusion matrices of cross-correlation in measured responses and simulated ones. A| Confusion matrices for an example cell, the same example cell used throughout. Each pixel represents the mean pairwise cross-correlation across responses to a training texture (abscissa) and test texture (ordinate) at a resolution of 2 ms. Correlation values scale with firing rate in Poisson neurons and, to a much lesser extent, in measured responses to texture. For the measured responses, correlation values for responses to the same training and test texture (in the diagonal) are higher than mismatched pairs, whereas in the Poisson model, no such structure exists. B-D| Same convention as in panel A with mean values across all cells (B), a subpopulation of PC-like cells (C), and a subpopulation of RA-like cells (D).

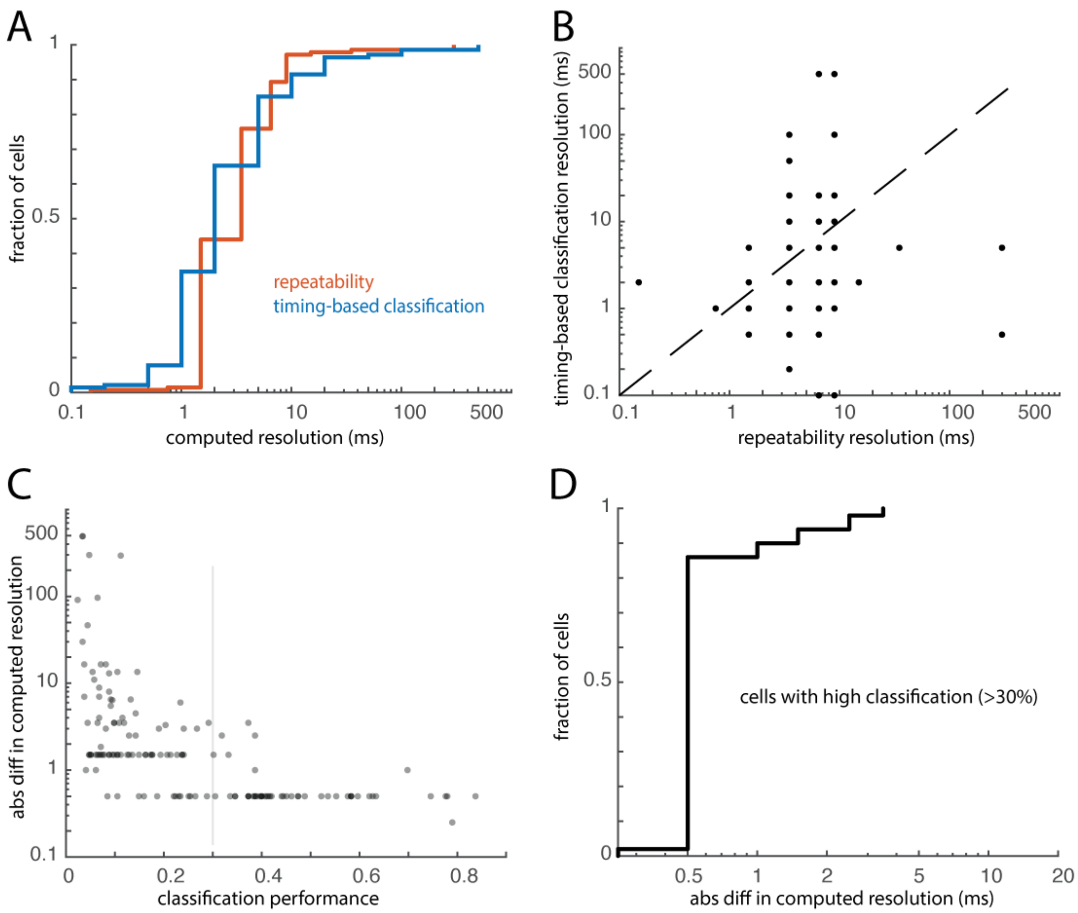

Supplemental Figure 4: Temporal resolutions derived from the repeatability and classification analyses. A| Cumulative distributions of resolutions obtained in the repeatability analysis (red) and those yielding maximum performance in the timing-based classification (blue). B| Comparison of the resolution determined from the repeatability analysis and timing-based classification. Each point represents one neuron; the dashed line denotes unity. C| Absolute difference in resolution between repeatability analysis and timing-based classification as a function of each neuron's classification performance. D| In neurons with a classification performance better than 30% (those to the right of the grey line in C), the resolution estimated from the timing-based classification is always within 5 ms of the resolutions estimated from the repeatability analysis, and often within 0.5 ms.

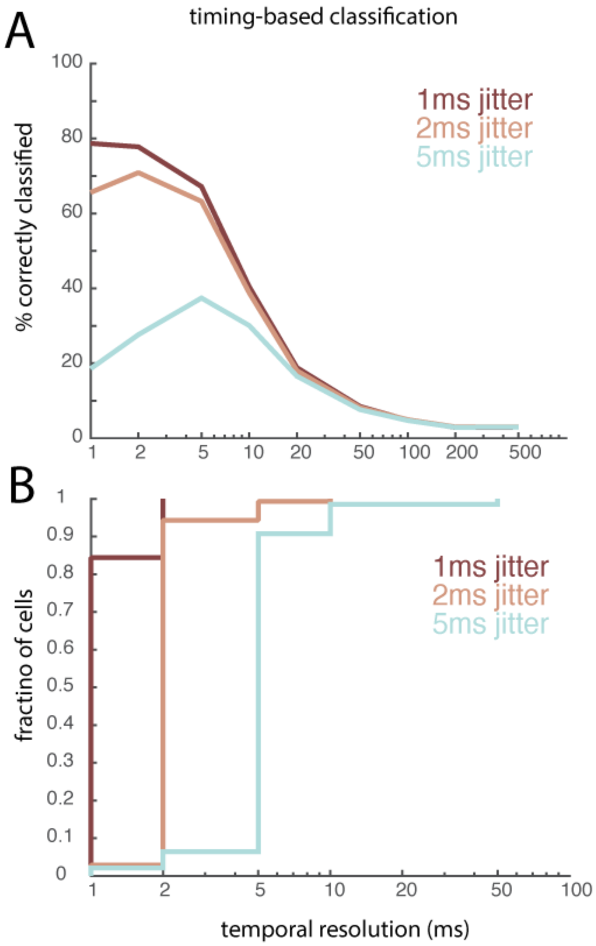

Supplemental Figure 5: Timing-based classification using simulated jittered responses. A| Less jitter in simulated spike trains results in better timing-based classification performance; Kruskal-Wallis test comparing max classification performance for each simulated cell across the three lowest levels of imposed jitter (χ^2= 142.6, p < 0.0001); post-hoc 1-sided Mann-Whitney U tests yield significant differences between 1-ms jitter (maroon) and 2-ms jitter (salmon, z-statistic = 4.0, p < 0.0001) and between 2-ms jitter and 5-ms jitter (blue, z-statistic = 9.2, p < 0.0001) . B| The best classification resolution coincides with the amount of imposed jitter in these simulated responses.

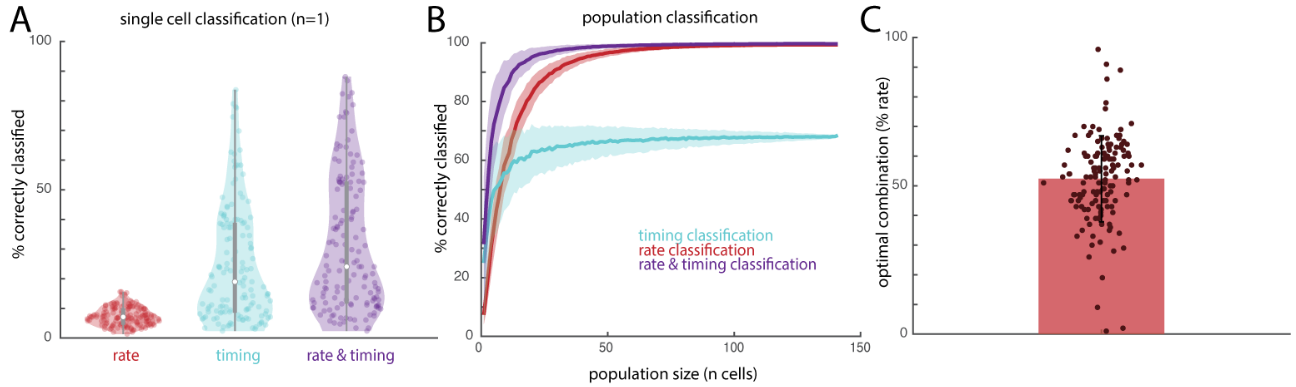

Supplemental Figure 6: Single-cell and population classification using rate, timing, and their mean. A| Single cell classification performance using rate difference, timing maximum cross-correlation, and an average of both (rather than an optimal combination). Violin plots show all values. Boxplots indicate median (center), interquartile range (boxes), and maximum and minimum (whiskers). B| Population classification using rate dissimilarity (red), timing cross-correlation (blue), and both (rather than an optimal combination, purple), averaged across the full neuronal population. Lines indicate the mean across 1000 iterations. Shaded areas denote standard deviation. C| The weighting of rate and timing that yields the highest classification performance for each cell. Each point denotes a single cell's optimal rate contribution. Error bars denote the standard deviation across 141 cells. The mean optimal weighting is 52.4%, which explains why both A and B (in which all cells' weightings are set to 50%) are nearly indistinguishable from Figure 2B and C (in which weighting is optimized for each cell).

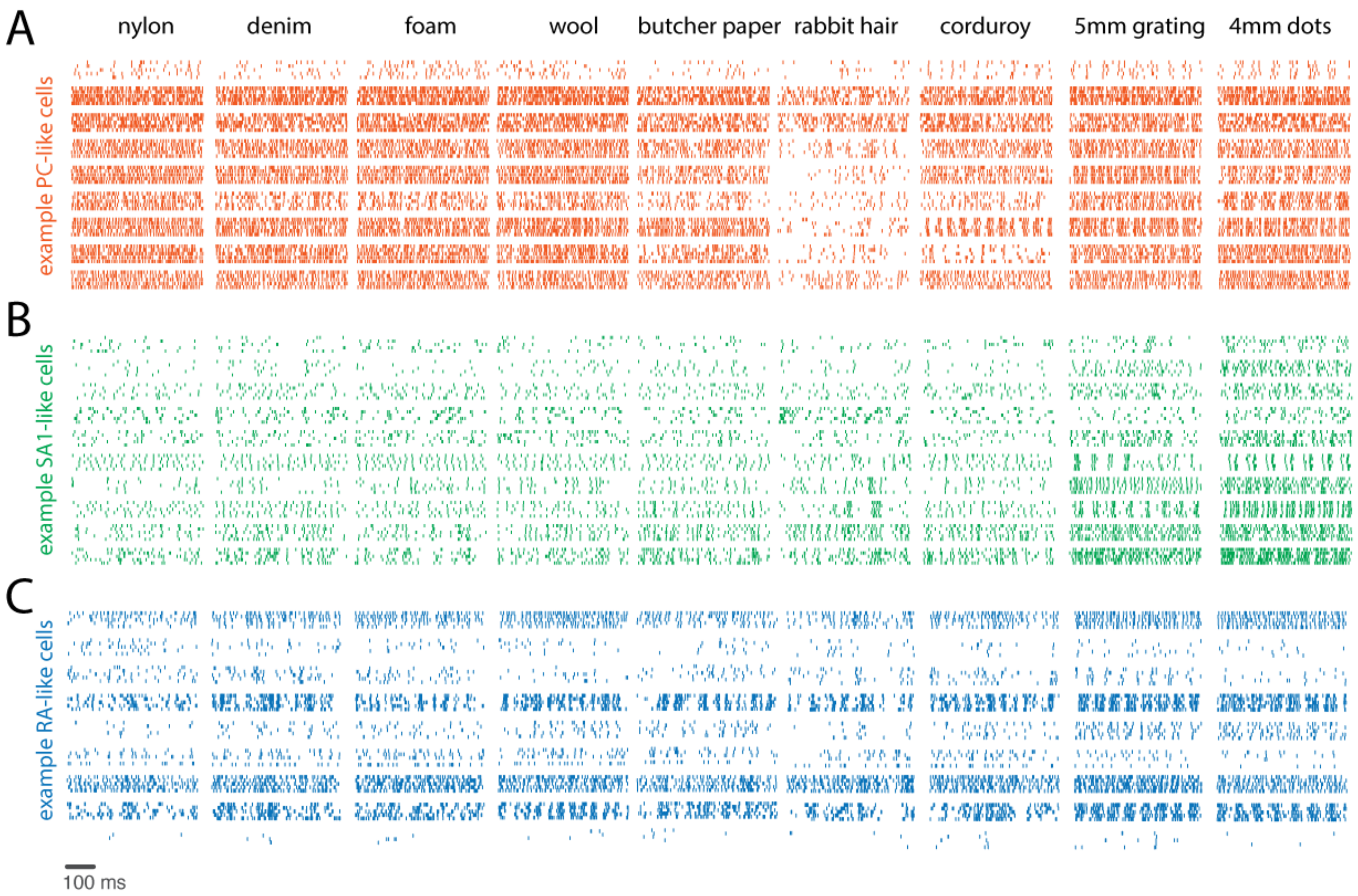

Supplemental Figure 7: Responses of PC-like, SA1-like, and RA-like cortical cells. A| Responses of nine example PC-like neurons to five repeated presentations of nine (of 59) textures. Each row is the response of an individual neuron across five repeated presentations of a given texture. B| Same as above, but for nine example SA1-like neurons. C| Same as above, but for nine example RA-like neurons.

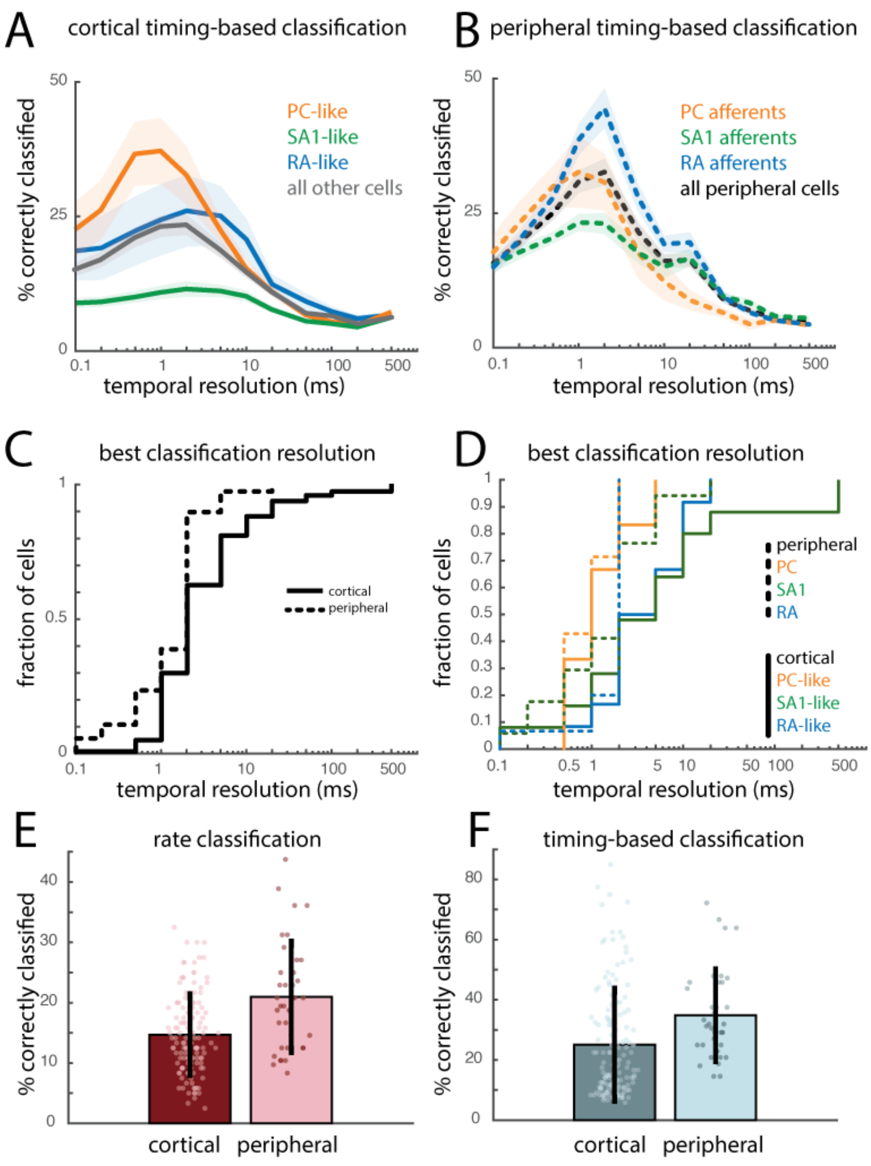

Supplemental Figure 8: Timing-based classification based on peripheral and cortical responses for a matched texture set. A| Timing-based classification performance across temporal resolutions for PC-like (orange), SA1-like (green), RA-like (blue), and all remaining unclassified neurons (grey) for a subset of 24 textures that were used in both the cortical and peripheral experiments. Shaded area denotes the standard error across cells. B| Timing-based classification performance for the same 24 textures based on PC (orange), SA1 (green), and RA (blue) afferent responses. Shaded area denotes the standard error across cells. C| The best classification resolutions are similar for cortical and peripheral neurons, but some cortical neurons are temporally imprecise whereas no afferents are. D| The temporal resolutions of PC fibers (orange dashed) and PC-like cortical neurons (orange solid) overlap, whereas some SA1-like (green solid) and RA-like cortical neurons (blue solid) are less precise than their afferent counterparts (green dashed and blue dashed, respectively). E| Single-cell classification based on firing rates evoked in cortical and peripheral neurons. Each point represents one neuron's classification performance. Bars indicate means and error bars denote standard deviation across 141 cortical and 39 peripheral neurons. Darker bar denotes cortical cells. F| Same as in E, but for timing-based classification.

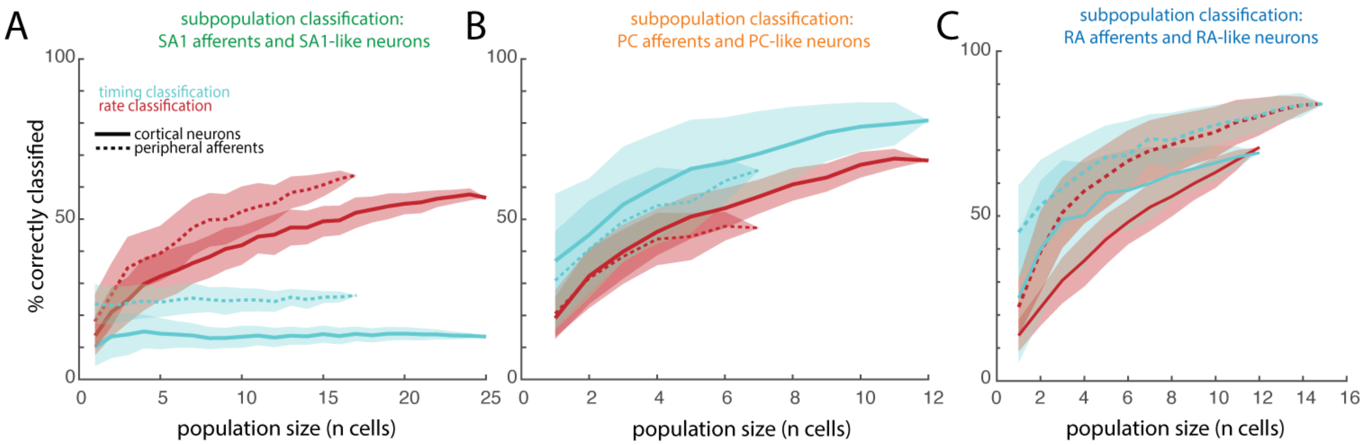

Supplemental Figure 9: Classification of textures based on peripheral and cortical population responses. A| Classification of 24 textures (chance = 1/24, 4.2%) based on the rate (rate) and timing (blue) of the response of populations of SA1 fibers. Shaded area denotes the standard deviation across 100 randomly sampled populations of peripheral or cortical neurons. Solid lines represent SA1-like cortical cells (*n* = 1-25); dashed-lines represent SA1 afferents in the peripheral nerve (*n*=1-17). B| Same, with PC-like cortical cells (*n*=1-12) and PC afferents (*n*=1-7). Shaded area denotes the standard deviation across 100 randomly sampled populations of peripheral or cortical neurons. C| Same, with RA-like cells (*n*=1-12) and RA afferents (*n*=1-15). Shaded area denotes the standard deviation across 100 randomly sampled populations of peripheral or cortical neurons.

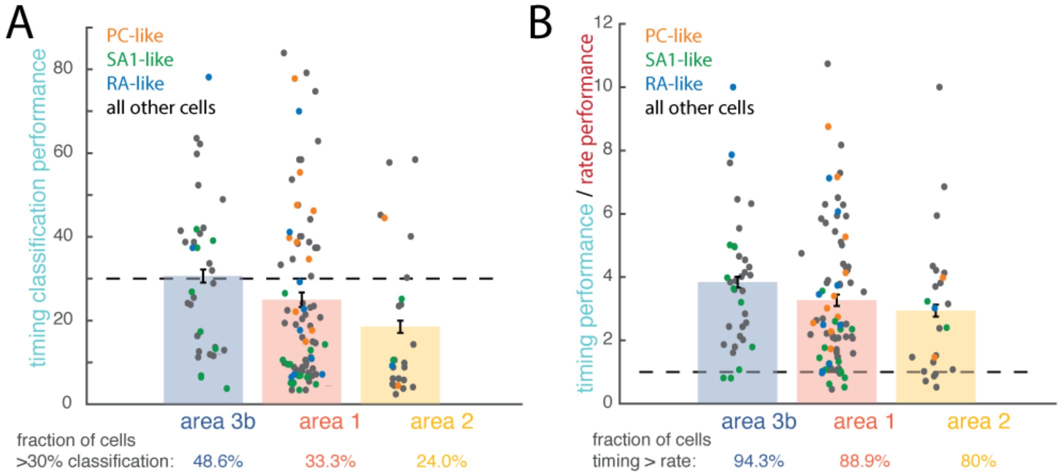

Supplemental Figure 10: Differences in temporal resolution across cortical fields. A| Single-cell timing-based classification performance across different cortical fields. Each circle represents one neuron's performance; orange, green, and blue circles are color-coded based on the neuron's dominant submodality input (PC, SA1, and RA, respectively). Bars indicate means and error bars denote the standard error across cells (n = 35 in area 3b, 81 in area 1, and 25 in area 2). B| Ratio of timing performance / rate performance for the same cells. Same conventions as in panel A. Dashed line represents equal performance for rate- and timing-based classifiers. Points above the line denote neurons for which timing outperforms rate.

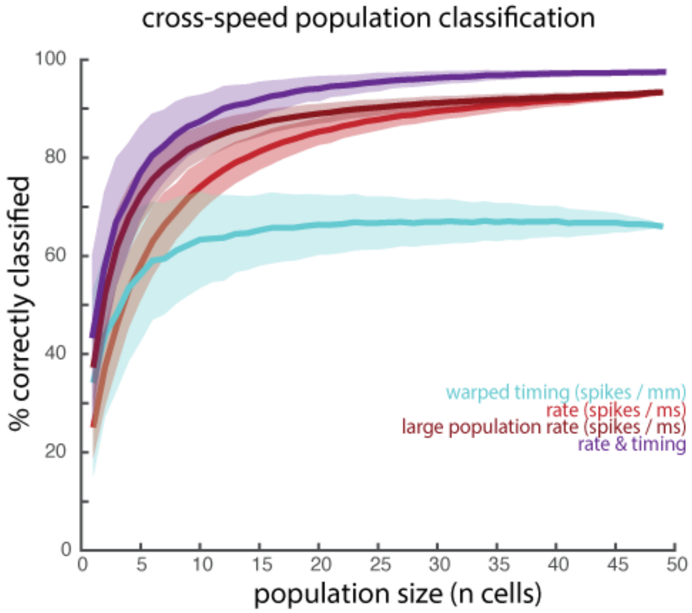

Supplemental Figure 11: Cross-speed classification based on both rate and timing outperforms classification based on rate. Mean cross-speed population classification based on rate (unwarped, red), timing (warped, blue), and the combination of both (purple). As a control, we averaged rate over a population twice the size (large population rate classification, maroon) to test the extent to which the improved performance with rate and timing was simply driven by more predictors. Performance from the large population never exceeds that of rate and timing. Shaded regions denote standard deviation across 1000 iterations of randomly sampled populations of 49 neurons.

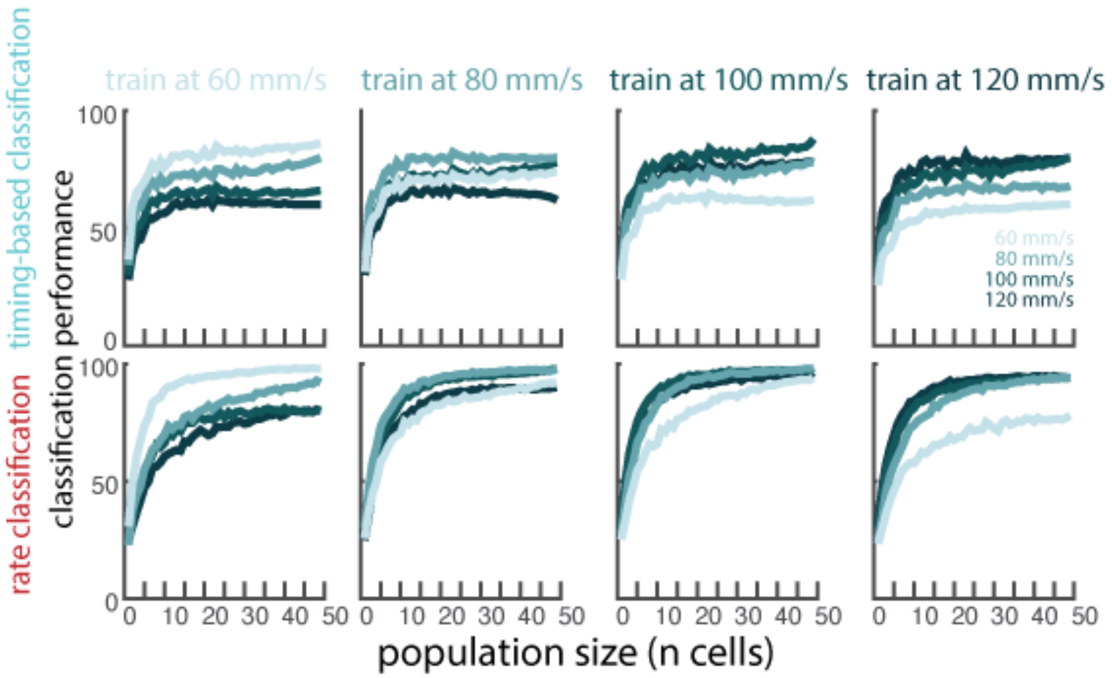

Supplemental Figure 12: Population classifiers trained on responses to textures presented at one speed and tested on responses to textures at all speeds. Timing-based classification with warped spike trains (top row) and rate-based classifiers with unwarped spike trains (bottom row), trained and tested at all combinations of speeds (light blue – dark blue, 60-120 mm/s).

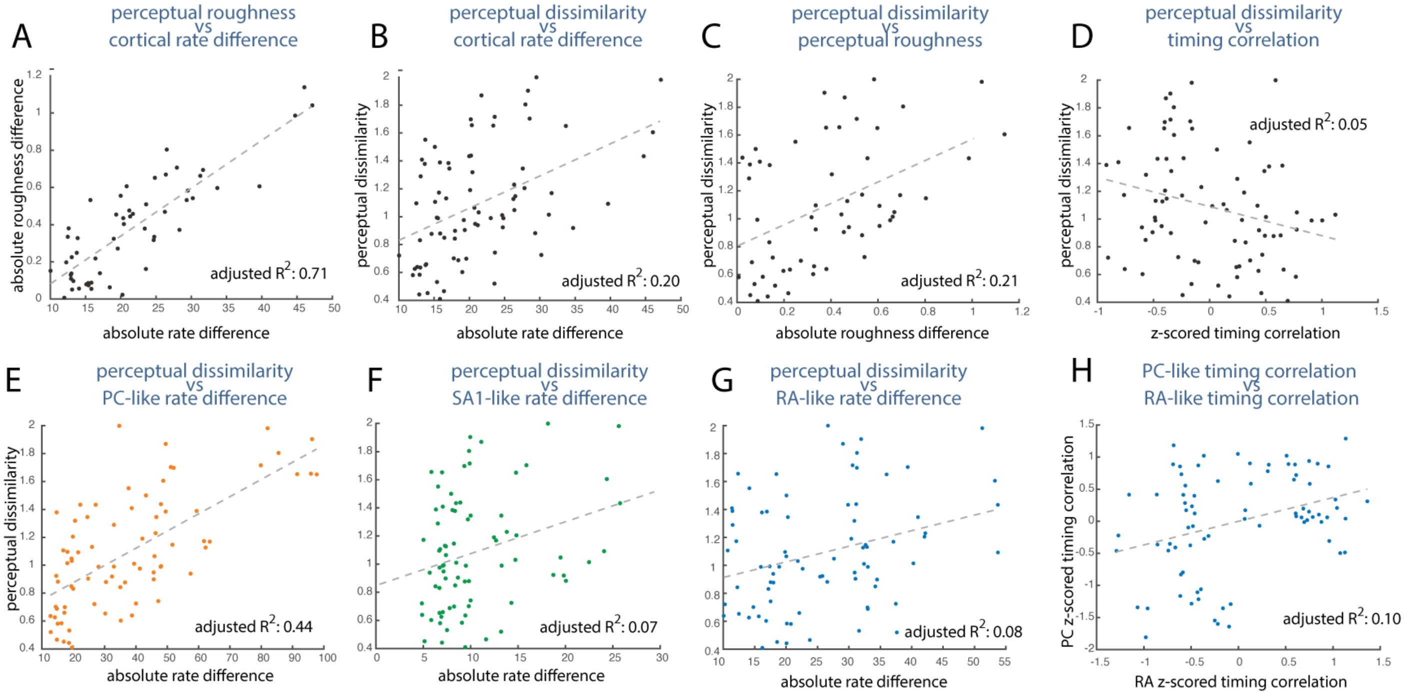

Supplemental Figure 13: Roughness, but not dissimilarity, is predicted by firing rate. A| The absolute difference in mean firing rate across the full population of 141 cortical cells vs. the absolute difference in roughness ratings (rated by 6 human subjects) for the same texture pairs (78 unique pairs). Differences in firing rate predict differences in perceived roughness. B| Differences in firing rate only account for 20% of the variance in the dissimilarity ratings (rated by 10 human subjects). C| Similarly, differences in roughness account for 20% of the variance in dissimilarity ratings. Thus, many textures are similarly rough but feel very different nonetheless. D| Mean z-scored timing correlation across the full population of 141 cortical neurons is a poor predictor of dissimilarity. E| Although firing rate differences across all neurons do not correlate with perceived dissimilarity (as in C), firing rate differences in a population of 12 PC-like cortical cells do. F-G| Mean firing rate differences in a population of 25 SA1-like neurons (F) or 82 RA-like neurons (G) are both poor predictors of dissimilarity ratings. H| Although RA-like cells demonstrate temporal reliability, timing correlations among RA-like cells are different from their PC-like counterparts.

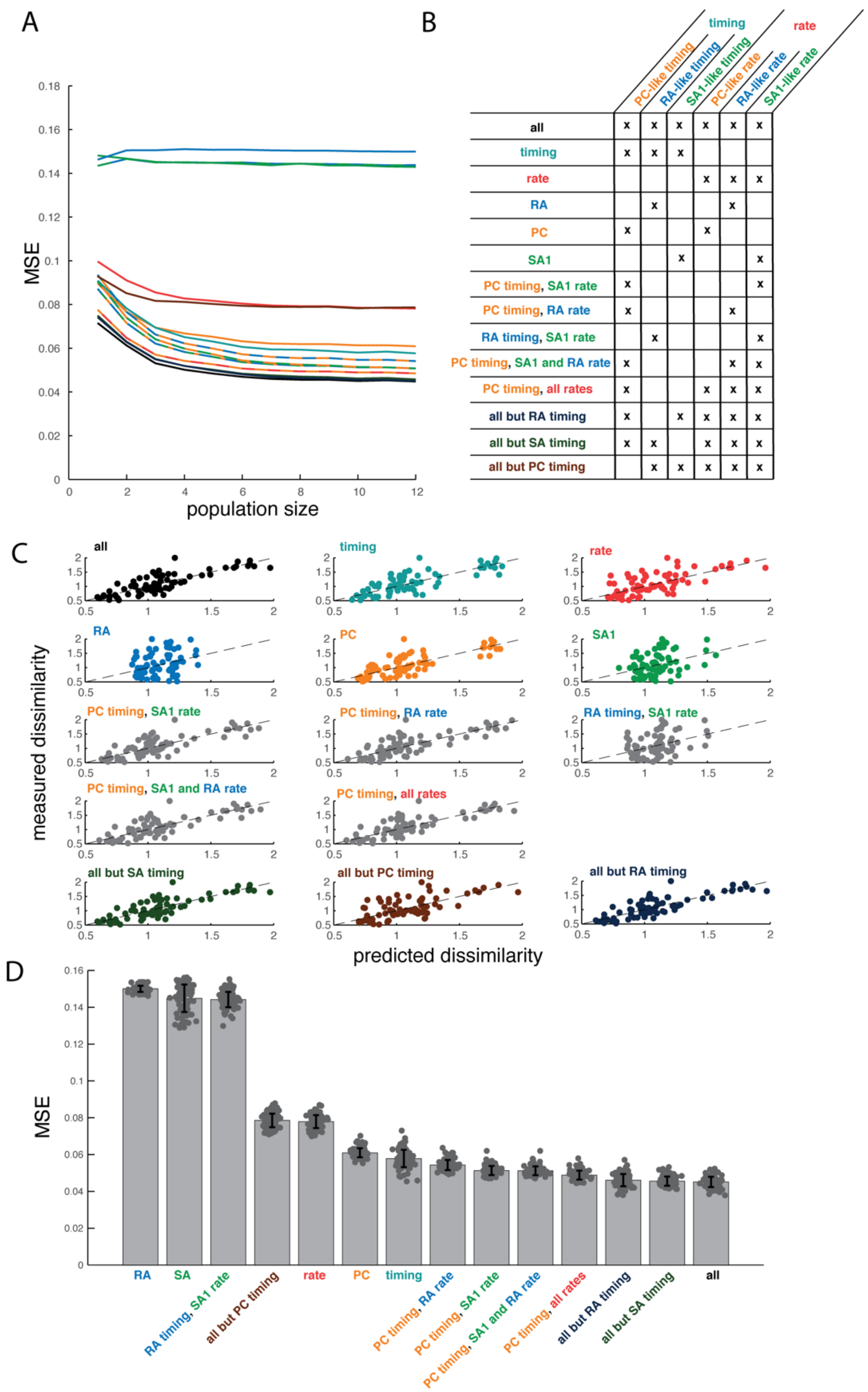

Supplemental Figure 14: Multi-factor multiple regression onto perceived dissimilarity of texture pairs. A| Cross-validated mean squared error (MSE) of the predicted dissimilarity vs. population size. Each colored trace corresponds to one of the models, listed in panel B. B| Factors included in the multiple regression. The "all" model in black includes six factors: rate and timing from all submodality types (PC-like, SA1-like, RA-like). The rate and timing model includes three factors: either rate or timing from each of the submodality types. The remaining models each include the factors indicated. C| Predicted vs. measured dissimilarity for all 78 texture pairs using the regression models shown in panels A-C with samples of 10 neurons. Dashed lines denote unity. D| MSE for every iteration of every model, each with a different sample of 10 neurons. Models with PC timing and rate perform as well as the model with all predictors included. Bars indicate the mean across 100 iterations. Error bars denote standard deviation.

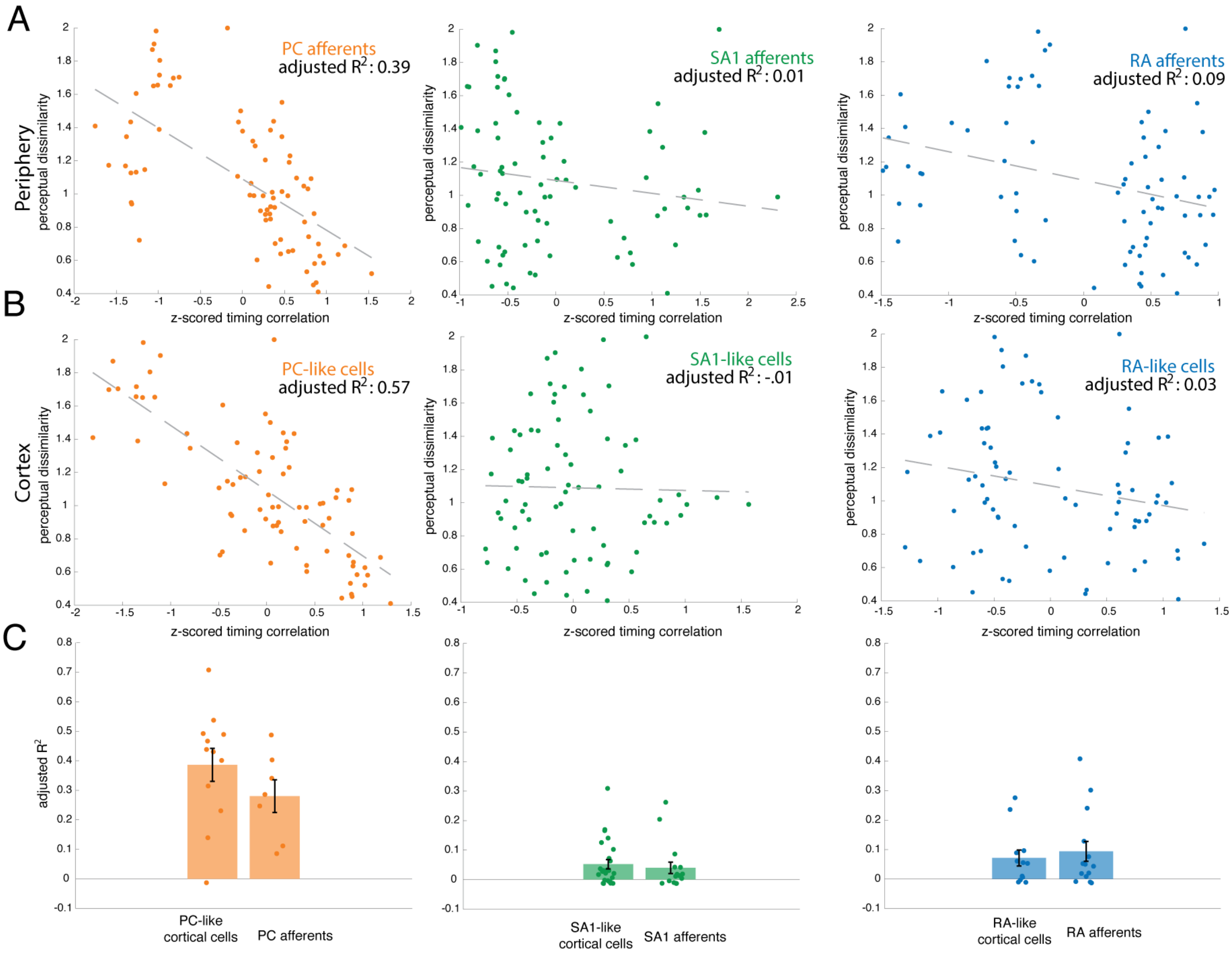

Supplemental Figure 15: Temporal patterns in PC and PC-like responses more closely reflect perceptual ratings than do their SA1 or RA counterparts. Regression of timing correlation on perceived dissimilarity. Each point represents one texture pair. Dashed lines represent the best linear fit. A| Peripheral afferents. B|PC-like, SA1-like, and RA-like cortical cells. C| Adjusted R^2 values (as in panels A and B) for each individual PC-like cortical cell (n = 12, left, orange) and PC afferent (n = 7, right, orange), SA1-like cortical cell (n = 25, left, green) and SA1 afferent (n = 17, right, green), and RA-like cortical cell (n = 12, left, blue) and RA afferent (n = 15, right, blue). Bars indicate means and error bars denote standard error.

All textures	Shared with peripheral data	Dissimilarity texture set	Speed set
Chiffon	X	X	X
Nylon	X	X	X
Stretch Denim	X		X
Hucktowel	X	X	X
City Lights			X
Deck Chair (Sunbrella)			X
Dots / 1 Grating			X
Fuzzy Upholstery (Blue)			X
Dots / Blank			X
Faux Croc Skin			X
Onyx Pavillion (Upholstery)			
Grid Upholstery			
Red Grating Upholstery			
Green Upholstery			
Unknown Pleasures			
Wrapping Paper (Bumpy)			
Crinkled Silk	X	X	
Parchment Paper			
20 Gauge Vinyl	X		
Microsuede	X	X	
Sueded Cuddle (Suede Side)	X	X	
Foam (Drapery Tape)	X		
5/500 Grating			
5/1 Grating			
Wool Blend	X	X	
Denim	X	X	
Satin	X	X	
Wool Gabardine	X		
Metallic Silk	X	X	
Velvet	X		
Computer Paper			
Careerwear Flannel	X		
Butcher Paper			
Organza	X		
Ruby Dots (Front)			
Yellow Upholstery		X	
Beach Mat (Tan)			
Corduroy - Thick Ridges	X	X	
Leathery Dots			
Long Hair Rabbit			
Short Hair Rabbit			
Snowflake Fleece (Fuzzy Side)	X		
20 Percent Wool Felt	X		
Possum Fur			
Sting Ray Skin			
Blank - Acrylic			
5 mm Grating	X		
1 mm Grating	X		
500 micron Grating			
RF 1			
Black Fabric Grating		X	
Embossed Dots - 4mm	X		
Embossed Dots - 5mm	X		
Dots / 500 Grating			
RF 2			
Tan Upholstery			
Lizard Skin			
Bumpy Polyester			
Ruby Dots (Backing)			

Supplemental Table 1: Texture set for each experiment. Column 1 includes all 59 textures presented during cortical neurophysiological recordings. Some of these textures had also been used in previous experiments: Column 2 shows the 24 textures that overlapped with those used in the peripheral experiments. Column 3 shows the 13 textures used in the psychophysical experiments. Column 4 shows the 10 textures that were presented at 4 speeds (60, 80, 100, 120 mm/s).
