# Supplementary material for: Texture is encoded in precise temporal spiking patterns in primate somatosensory cortex
Source: Nat Commun. 2022 Mar 14;13:1311. doi: 10.1038/s41467-022-28873-w (PMC8921276; doi:10.1038/s41467-022-28873-w)
Supplement: Supplementary file 3 — Reporting Summary [file 41467_2022_28873_MOESM3_ESM.pdf]

nature research

Corresponding author(s): Katie Long, Justin Lieber, Sliman Bensmaia

Last updated by author(s): 01/12/2022

Reporting Summary

Nature Research wishes to improve the reproducibility of the work that we publish. This form provides structure for consistency and transparency in reporting. For further information on Nature Research policies, see our Editorial Policies and the Editorial Policy Checklist.

Statistics

For all statistical analyses, confirm that the following items are present in the figure legend, table legend, main text, or Methods section.

n/a	Confirmed	
☐	☒	The exact sample size (n) for each experimental group/condition, given as a discrete number and unit of measurement
☐	☒	A statement on whether measurements were taken from distinct samples or whether the same sample was measured repeatedly
☐	☒	The statistical test(s) used AND whether they are one- or two-sided<br>*Only common tests should be described solely by name; describe more complex techniques in the Methods section.*
☐	☒	A description of all covariates tested
☐	☒	A description of any assumptions or corrections, such as tests of normality and adjustment for multiple comparisons
☐	☒	A full description of the statistical parameters including central tendency (e.g. means) or other basic estimates (e.g. regression coefficient) AND variation (e.g. standard deviation) or associated estimates of uncertainty (e.g. confidence intervals)
☐	☒	For null hypothesis testing, the test statistic (e.g. F, t, r) with confidence intervals, effect sizes, degrees of freedom and P value noted<br>*Give P values as exact values whenever suitable.*
☒	☐	For Bayesian analysis, information on the choice of priors and Markov chain Monte Carlo settings
☒	☐	For hierarchical and complex designs, identification of the appropriate level for tests and full reporting of outcomes
☐	☒	Estimates of effect sizes (e.g. Cohen's d, Pearson's r), indicating how they were calculated

Our web collection on statistics for biologists contains articles on many of the points above.

Software and code

Policy information about availability of computer code

Data collection	Neurophysiological data were collected using a tungsten electrodes (FHC Inc) and a computer-controlled multielectrode microdrive (NAN Instruments). Signals were recorded using a PBX3 amplifier (Plexon Inc) and an analog to digital converter (National Instruments Corp). Human psychophysical data were recorded by MATLAB protocols that prompted responses from human subjects.
Data analysis	All analyses were performed using MATLAB (R2019b) and all code can be found on GitHub (https://github.com/kthlong/TimingAnalyses)

For manuscripts utilizing custom algorithms or software that are central to the research but not yet described in published literature, software must be made available to editors and reviewers. We strongly encourage code deposition in a community repository (e.g. GitHub). See the Nature Research guidelines for submitting code & software for further information.

Data

Policy information about availability of data

All manuscripts must include a data availability statement. This statement should provide the following information, where applicable:

- Accession codes, unique identifiers, or web links for publicly available datasets
- A list of figures that have associated raw data
- A description of any restrictions on data availability

All data are available on GitHub (https://github.com/kthlong/TimingAnalyses).

Field-specific reporting

Please select the one below that is the best fit for your research. If you are not sure, read the appropriate sections before making your selection.

☒ Life sciences ☐ Behavioural & social sciences ☐ Ecological, evolutionary & environmental sciences

For a reference copy of the document with all sections, see nature.com/documents/nr-reporting-summary-flat.pdf

Life sciences study design

All studies must disclose on these points even when the disclosure is negative.

Sample size	We recorded from four hemispheres in two separate animals. We recorded from 141 single units in somatosensory cortex of two different non-human primates. We also measured psychophysical responses from 16 human subjects.
Data exclusions	We only included neurons with good isolation over background noise and whose receptive fields were centered on the distal pads of digits 2-5. Any other cells encountered during mapping were not used. All data from human subjects were included.
Replication	All classification analyses and regression analyses were cross-validated by training on 4 repeats and testing on the 5th, left-out repeat. This was repeated until each unique repetition was used as the test repeat.
Randomization	Randomization was not required for our analyses, because we recorded any neuron that met the criteria listed above and did not group cells for particular experiments. We presented the same set of stimuli to all recorded cells for as long as a stable recording was maintained.
Blinding	We did not characterize the submodality input of cells until after all data had been collected and therefore did not introduce bias in the nature of cells we chose to record. No further blinding was necessary, as stimuli were presented pseudorandomly in both the psychophysical and neurophysiological experiments to prevent any biases.

Reporting for specific materials, systems and methods

We require information from authors about some types of materials, experimental systems and methods used in many studies. Here, indicate whether each material, system or method listed is relevant to your study. If you are not sure if a list item applies to your research, read the appropriate section before selecting a response.

Materials & experimental systems

n/a	Involved in the study	
☒	☐	Antibodies
☒	☐	Eukaryotic cell lines
☒	☐	Palaeontology and archaeology
☐	☒	Animals and other organisms
☐	☒	Human research participants
☒	☐	Clinical data
☒	☐	Dual use research of concern

Methods

n/a	Involved in the study	
☒	☐	ChIP-seq
☒	☐	Flow cytometry
☒	☐	MRI-based neuroimaging

Animals and other organisms

Policy information about studies involving animals; ARRIVE guidelines recommended for reporting animal research

Laboratory animals	This study relied on data collected from two male rhesus macaques (Macaca mulatta), ages 6-8 years old and weighing 8-11 kg.
Wild animals	This study did not involve wild animals.
Field-collected samples	This study did not involve field-collected samples.
Ethics oversight	All experimental procedures involving animals were approved by the University of Chicago Institutional Animal Care and Use Committee (ACUP 72042).

Note that full information on the approval of the study protocol must also be provided in the manuscript.

Human research participants

Policy information about studies involving human research participants

Population characteristics	This study relied on psychophysical data collected from 16 human subjects, (dissimilarity ratings: 10 females, ages 19-24; roughness ratings: 5 males and 1 female, ages 18-24).

Recruitment	Subjects were paid volunteers recruited through physical flyers around the University of Chicago campus. This method of recruitment is highly unlikely to bias our results in any way.
Ethics oversight	All human psychophysical protocols were approved by the Institutional Review Board at the University of Chicago (IRB 15-1670).

Note that full information on the approval of the study protocol must also be provided in the manuscript.
